# Effect of space allowance and transport container height on the welfare of fattening rabbits under different environmental thermal conditions

**DOI:** 10.3389/fvets.2025.1658548

**Published:** 2025-09-02

**Authors:** A. Contreras-Jodar, A. Dalmau, M. Bagaria, J. A. D. Barbosa-Filho, M. Rendon, A. A. K. Salama, A. Velarde

**Affiliations:** ^1^IRTA, Animal Welfare, Monells, Spain; ^2^Department of Agri-Food Engineering and Biotechnology, Universitat Politècnica de Catalunya, Castelldefels, Spain; ^3^Department of Agricultural Engineering, Universidade Federal Do Ceará, Fortaleza, Brazil; ^4^Ruminant Research Group (G2R), Department of Animal and Food Sciences, Facultat de Veterinària, Universitat Autònoma de Barcelona, Bellaterra, Spain

**Keywords:** rabbit, welfare, transport, space allowance, stocking density, height, thermal stress, thermal imaging

## Abstract

This study evaluated the combined effects of container space allowance, height, and ambient thermal conditions on the welfare of fattening rabbits during simulated transport. Nine hundred rabbits were exposed to three space allowances (121, 150, and 191 cm^2^/kg; S121, S150, S191), two container heights (20 and 35 cm; H20, H35), and four thermal environments (21.4 °C, 25.9 °C, 30.0 °C, and 33.5 °C, T1–T4, all at 50–68% RH) for 8 h after 6-h fasting. Welfare was assessed via thermophysiological (rectal temperature, RT; body weight loss, BWL) and metabolic indicators (hematocrit, glucose, LDH, corticosterone, creatine kinase, NEFAs). Thermographic imaging of ear and lacrimal regions was correlated with RT to validate a non-invasive method for assessing thermal stress. T4 was stopped after 5 h due to severe compromise in S121 and S150, especially in H35. T1 increased hypothermia risk compared to T2 and T3 (*p* = 0.043). S191 increased hypothermia risk compared to S150 and S121. Cage height did not affect hypothermia (*p* = 0.875) but increased hyperthermia risk under T3, especially in S150 and S121 (*p* < 0.037). BWL varied with thermal state (*p* < 0.001): 54.4 g in hypothermia, 65.2 g in normothermia, 74.1 g in hyperthermia. RT correlated with lacrimal (r = 0.743) and ear (r = 0.704) temperatures (*p* < 0.001). Hematocrit, LDH, and creatine kinase remained stable. Glucose varied with space allowance (*p* = 0.002) and tended to vary with height (*p* = 0.070), highest in S121 and H20. NEFAs decreased under T3 (*p* < 0.010) and tended to under T2. These findings highlight the importance of optimizing transport conditions and support thermography as a welfare monitoring tool.

## Introduction

Approximately 74 million rabbits were transported from farms to slaughterhouses in the European Union (EU) in 2024 (European Rabbit Association, personal communication). Rabbit farming for consumption is mainly concentrated in three Member States, which account for 84.2% of the EU’s total production. Spain is the leading producer (39.6%), followed by France (25.2%) and Italy (19.4%). The remaining 15.8% of production is mainly distributed among Hungary, Portugal, the Netherlands, Poland, the Czech Republic, and Belgium (European Rabbit Association, personal communication).

The transport of rabbits within the same Member State for slaughter accounts for the majority of journeys, and 99% of transport is carried out by road (1). Transport time is usually under 4 h in all EU countries, with some exceptions (e.g., rerouting to another slaughterhouse due to flooding), where it can be up to 8 h (European Rabbit Association, personal communication). This time does not include the time spent on capture, loading, and unloading of the rabbits.

Currently, in the EU, the commercially used space allowance for the transport of fattening rabbits ranges between 111 and 167 cm^2^/kg, with the most commonly used value in practice being around 143 cm^2^/kg. This corresponds to a loading density of between 60 and 90 kg/m^2^, with 70 kg/m^2^ being the most commonly applied (European Rabbit Association, personal communication), and container heights varying between 20 and 30 cm (2). So far, this heterogeneity is due to the lack of specific requirements in the current EU legislation, which does not establish minimum standards for these variables in the road transport of rabbits.

According to EFSA (1), the minimum space allowance per rabbit should correspond to the area occupied by a rabbit lying in a resting posture, calculated using the allometric formula proposed by Petherick and Phillips (3) [available space (cm^2^/rabbit) = 270 × live weight (kg^2/3^)]. Additionally, EFSA (1) reported that the height of the containers should allow rabbits to sit in a natural upright posture without their ears touching the ceiling of the container. Otherwise, they may experience movement restrictions and be unable to stretch their ears, which would impair their ability to dissipate heat under thermally challenging conditions. However, the report acknowledges that it remains unclear whether rabbits would actually adopt this upright sitting posture on their hind legs during transport, even if sufficient space was available.

As a guideline, EFSA (1) suggested that the space allowance for slaughter rabbits of commercial live weight (2.0–2.5 kg) should not exceed 200–215 cm^2^/kg (equivalent to a stocking density of 47–50 kg/m^2^). This is slightly lower than the area a rabbit occupies in lying position, which has been quantified at 176–194 cm^2^/kg or 52–57 kg/m^2^ by Giersberg et al. (4). However, the space allowance required for all rabbits within a transport container to lie down simultaneously was estimated at 166 cm^2^/kg or at a stocking density of 60 kg/m^2^ (5). The recommended container height according to EFSA (1) was at least 35 cm for rabbits up to 3 kg.

EFSA (1) also identified and described the main welfare consequences for rabbits during transport. These include prolonged hunger and thirst, restriction of movement within transport containers, thermal stress, and motion stress.

Stress caused by prolonged hunger and thirst is a common experience across all transported animals, as it is inherent to the process itself. Nevertheless, as with other species, rabbits are subjected to a pre-transport fasting period (1, 6, 7). Withholding feed prior to slaughter helps reduce the amount of gastrointestinal content, thereby lowering the risk of carcass contamination. Furthermore, fasting before and during transport helps minimize the amount of feces in the vehicle, which can enhance the efficiency of the transport process (7).

In the case of slaughter rabbits, fasting includes both feed and water withdrawal for 4 to 6 h prior to capture and loading onto the vehicle. Unlike other species, rabbits are not only deprived of feed but also of water, since if they are allowed to drink but not eat, they may develop diarrhea during transport (European Rabbit Association, personal communication).

Restriction of movement also affects all transported rabbits, as the available space and height within transport containers are limited. The extent of this restriction depends on whether the space allowance and container height enable the animal to adopt natural postures, move and rest comfortably. However, very few scientific studies have evaluated the effect of available space on rabbit welfare (8–10), and to our knowledge, none have addressed the impact of container height.

Thermal stress, whether due to heat or cold, can compromise homeostasis in rabbits by impairing their capacity to regulate body temperature. Exposure to temperatures outside the comfort zone not only impairs animal welfare but may also trigger physiological and metabolic responses, potentially resulting in dehydration, body weight loss, and defects in meat quality. If the animal is unable to cope with thermal stress, death may occur (11).

Motion stress refers to the experience of motion sickness, stress, and/or fatigue as a result of acceleration, braking, stops, turns, gear shifts, vibrations, noise, and uneven road surfaces during transport. It is well known that vibrations, accelerations, and impacts can result in poor postural stability, muscle fatigue, exhaustion, and, in some cases, motion sickness (1).

Following the EFSA report (1), in December 2023, the European Commission presented a proposal to revise Council Regulation (EC) No. 1/2005, with the aim of improving animal welfare during transport and aligning the legislation with best practices in animal welfare management. However, rabbits remain one of the least-studied species in this context, and scientific evidence on the necessary space and appropriate container height, particularly in relation to ambient temperature and humidity during transport, is still lacking.

Therefore, the objective of this study was to evaluate the combined effects of prolonged hunger and thirst and movement restriction under varying ambient temperature, using a range representative of typical Mediterranean conditions, where approximately 84.2% of rabbit transport to slaughterhouse in the EU takes place. Specifically, the study aims to assess the impact on the welfare of fattening rabbits of two heights (20 cm which is the minimum container height and the most commonly used within the EU, and 35 cm, as recommended by EFSA (1) to avoid vertical movement restriction) combined with three space allowance treatments (182, 143, and 111 cm^2^/kg, equivalent to 55, 70, and 90 kg/m^2^, respectively). These space allowance values represent the space needed for all rabbits to lie down simultaneously according to Giersberg et al. (4), the most commonly space allowance used in commercial transports within the EU, and the lower space allowance currently in use in commercial transport. These treatments were assessed under four environmental conditions of temperature and relative humidity. Welfare was evaluated through thermophysiological and metabolic indicators during 8 h simulated journeys in rabbits previously subjected to a standard commercial fasting period of 6 h.

Furthermore, considering that rabbits are a prey species with ground-dwelling habits, direct handling can trigger an acute stress response, potentially resulting in increased core body temperature. Therefore, this study also aimed to compare and validate thermographic imaging of the auricular pavilion and lacrimal region by correlating it with rectal temperature, with the objective of identifying a non-invasive, rapid, and less stressful method for assessing body temperature as an indicator of thermal stress following transport.

## Materials and methods

The animal care conditions and handling practices of the study were approved by the Ethics Committee for Animal and Human Experimentation (CEEAH) of the Universitat Autònoma de Barcelona (UAB) under protocol CEEAH 5682-CEEA-UAB on April 23, 2024.

### Animals, housing and treatments

A total of 900 fattening rabbits of both sexes, all of the same (hybrid) genetic line and homogeneous body weight (1.992 ± 0.041 kg), were transported from a commercial farm to Servei de Granges i Camps Experimentals (SGCE) of the UAB in Bellaterra (Barcelona, Spain) in ten batches of 90 rabbits from both sexes each, between September and December 2024.

Each batch was housed for three days to allow an acclimation period and to mitigate any residual transport stress. During this time, the animals were kept in slatted-floor pens with ad libitum access to feed (the same diet as provided on the farm of origin) and water.

Prior to each experimental session, the corresponding batch of rabbits underwent a 6 h feed and water withdrawal period to simulate commercial fasting conditions. Rabbits were then individually weighed (WA200, MeierBrakenberg, Brakenberg, DE) and immediately housed in groups of five rabbits per cage. A total of 18 cages were used per session, housed within a climate-controlled chamber (Carel Controls Ibérica, Barcelona, ES).

Six cage types were designed by combining three target space allowances: (1) 111 cm^2^/kg, equivalent to 90 kg/m^2^ (cage: 50 cm × 24 cm); (2) 143 cm^2^/kg, equivalent to 70 kg/m^2^ (cage: 50 cm × 30 cm); and (3) 182 cm^2^/kg, equivalent to 55 kg/m^2^ (cage: 50 cm × 38 cm), combined with two height levels: 20 cm (H20) or 35 cm (H35). However, due to the actual final body weight of the rabbits in the experiment, the realized space allowances were 121 cm^2^/kg, 150 cm^2^/kg, and 191 cm^2^/kg, respectively, rather than the intended values. Consequently, the space allowance treatments were designated as S121, S150, and S191 to reflect the adjusted measurements.

This design resulted in six cage types based on space allowance and height: S121-H20, S121-H35, S150-H20, S150-H35, S191-H20, and S191-H35, each with three replicates (i.e., a total of 18 cages).

The different cage dimensions were intended to induce varying degrees of movement restriction in rabbits, both in terms of area and height. Cages were elevated 2 cm above the ground using wooden blocks to prevent rabbits from coming into contact with feces and urine excreted during the experimental session.

The full experimental procedure was conducted over ten independent sessions, each comprising six cage types with three replicates per type and five rabbits per cage, resulting in a total of 900 rabbits.

The climate chamber was located in a room adjacent to the housing pens, with dimensions of 4 m (width) × 6 m (length) × 2.3 m (height). It was equipped with a temperature and humidity control system (Carel Controls Ibérica, Barcelona, ES). The chamber was also equipped with six video cameras (IP Camera DH-IPC-HDW2231TP-ZS-S2, Zhejiang Dahua Vision Technology Co., Ltd., Hangzhou, CN) connected to a digital video recorder (Network Video Recorder DHI-NVR4108-8P-4KS2/L, Zhejiang Dahua Vision Technology Co., Ltd., Hangzhou, CN). Each camera was positioned laterally and focused on three cages to enable continuous monitoring of rabbit behavior.

Six temperature and relative humidity sensors (HOBO MX2301 Temp/RH Data Logger, HOBO Data Loggers, Bourne, Massachusetts, United States) were used and programmed to automatically record data every minute. The sensors were evenly distributed throughout the chamber and placed near the cages at the height corresponding to the rabbits’ heads.

In each experimental session, the climate chamber was programmed to simulate four environmental conditions (thermal treatments) representative of truck transport over an 8 h period. The thermal treatments were: T1 (21.4 °C and 68.6% relative humidity; *n* = 3), T2 (25.9 °C and 58.4% relative humidity; *n* = 3), T3 (30.0 °C and 55.8% relative humidity; *n* = 3), and T4 (33.5 °C and 58.2% relative humidity; *n* = 1). Treatment T4 lasted 5 h instead of 8 h because rabbits began vocalizing and mortality was observed in some rabbits, which escaped the cameras’ view, especially in cages with the lowest space allowance (S121; 8 out of 30; 27%) so it was decided that this would be the endpoint criterion in animal experimentation. Consequently, T4 was executed only once, and its data was excluded from the analysis.

### Thermophysiological response

At the end of the 8 h thermal treatment, the presence of urine in the rabbits, evidenced by yellowish staining of the fur, presence of moisture, and a characteristic urine odor, was assessed and recorded. Subsequently, three animals per cage were randomly selected for rectal temperature measurement using a digital thermometer, followed by thermal imaging acquisition while still in the climatic chamber.

Thermographic images were captured using a FLIR-E64501 camera (Teledyne FLIR Systems Inc., Wilsonville, United States) with a resolution of 240 × 320 pixels, an 18 mm focal length lens, an accuracy of 0.03, and a thermal sensitivity range from −20 to 120 °C. Prior to use, the camera emissivity was set to 0.97, reflected temperature to 15 °C, distance to 1 m, and relative humidity and temperature were adjusted according to the environmental values for each treatment. Thermal images of three rabbits per cage were taken (54 rabbits per experimental day). For imaging, the animal was removed from its cage and placed on the floor at 0.5 m from the camera, measured with a ruler. Two photographs per rabbit were taken, oriented toward the left lateral side of the face, including the entire ears. Of the two images taken per animal, the one of best quality was selected and transferred to processing software (FLIR Tools v.6.4.18039.1003, FLIR Systems Inc., Wilsonville, United States). Maximum, minimum, and average temperatures of the ear and lacrimal regions were measured using the “circle” function.

After thermographic imaging, rabbits were weighed, sedated, and euthanized. Additionally, the other two animals per cage (36 rabbits per experimental day) were weighed, sedated, blood sampled by cardiac puncture, and euthanized.

For sedation, 0.5 mL of a mixture of xylazine 200 (at a dose of 40 mg/kg) and ketamine 100 (at a dose of 5 mg/kg) was used. A dose of 0.5 mL (xylazine + ketamine) per animal was administered intramuscularly using a 21G needle in the semitendinosus or semimembranosus muscle. After sedation, the rabbit’s status was verified by the absence of attempts to stand, corneal reflex, and spontaneous blinking before proceeding with blood collection. For blood sampling, 18G needles were used to extract approximately 5 mL of blood, which was transferred into two different tubes, one containing EDTA and one without EDTA. Euthanasia was then performed by injecting 0.8 mL of pentobarbital (Release^®^ injectable solution 300 mg/mL) into the animal’s heart.

### Metabolic response

Blood collected in tubes without EDTA was stored for at least 30 min at room temperature to allow coagulation, and subsequently centrifuged at 3000 rpm for 10 min. The serum corresponding to each rabbit was pipetted and transferred to an Eppendorf tube labeled with the same animal identification number to ensure sample traceability and stored at −20 °C until analysis by the Biochemistry Service and the Laboratory for Hormonal, Stress, Welfare, and Animal Reproduction Indicators at the UAB. The physiological markers analyzed were glucose (GLU), lactate dehydrogenase (LDH), creatine kinase (CK), non-esterified fatty acids (NEFAs), and corticosterone (CORT). Hematocrit (HCT) was analyzed from blood collected in EDTA tubes at the UAB Biochemistry Service.

GLU concentration was determined by enzymatic UV assay (hexokinase method; Beckman Coulter Reagent AU, Brea, CA, United States). LDH was measured using an enzymatic method (Olympus System Reagent^®^, Beckman Coulter^®^, Nyon, CH). CK was determined following the method recommended by the International Federation of Clinical Chemistry and Laboratory Medicine (IFCC) (Beckman Coulter Reagent AU, Brea, CA, USA). NEFAs were analyzed by a colorimetric enzymatic assay (ACS-ACOD) using a commercial kit (Wako Chemicals, Neuss, DE). CORT was measured by enzyme immunoassay using the commercial “Rabbit Corticosterone ELISA Kit” (FineTest, Wuhan, CN).

### Statistical analysis

All data were preprocessed, statistically analyzed, and graphically represented using R software version 4.3.2. All linear mixed models were fitted using the “nlme” package (12). In the mixed models, the marginal coefficient of determination (R^2^m) represents the proportion of the total variance explained solely by the fixed effects of the model. Conversely, the conditional coefficient of determination (R^2^c) reflects the proportion of variance explained by the complete model, including both fixed and random effects. The comparison between these values allows estimation of the relative contribution of random effects to the total variability of the outcome (13). Statistical significance was declared at *p* < 0.05 and trends at *p* < 0.10.

### Temperature and humidity conditions, initial body weight, and space allowance

Environmental temperature and relative humidity data obtained from the data loggers were analyzed using analysis of variance (ANOVA) to compare means among treatments, followed by multiple comparisons using the Tukey HSD method. Initial body weight of the rabbits was analyzed by ANOVA to compare means between treatments, with multiple comparisons performed using the Tukey HSD method. The actual space allowance was calculated by dividing the cage area by the sum of the initial body weights of all rabbits in the cage. Subsequently, it was analyzed by ANOVA with multiple comparisons using the Tukey HSD method.

### Thermophysiological response

Body weight loss (BWL) was calculated for each rabbit as the difference between initial body weight (after 6 h fasting) and prior to entering the climatic chamber and final body weight (after 8 h in the climatic chamber). For BWL and rectal temperature (RT), the experimental unit was the cage, and both variables were analyzed using a linear mixed model. Only thermal treatments with three replicates (T1, T2, T3), available space (S121, S150, S191), height (H20, H35), and their interactions were considered as fixed effects, while the experimental session (1 to 9) was included as a random factor.

Furthermore, according to the average RT rabbits per cage were classified into three thermal states: hypothermia (RT below 38.6 °C), normothermia (RT between 38.6 and 40.1 °C), and hyperthermia (RT above 40.1 °C), according to thresholds found in Kahn and Line (14). Subsequently, a multinomial logistic regression model was fitted using the “nnet” package (15) to evaluate the impact of thermal treatment, available space, and cage height on the rabbits’ thermal state. In this model, the dependent variable was the thermal state (categorized as hypothermia, normothermia, or hyperthermia), while the independent variables were thermal treatment, available space in the cage, and cage height. Predicted probabilities for each physiological state were calculated for all possible combinations of the predictor variables. Then, for each thermal treatment level, the combination of available space and height that maximized the probability of normothermia was selected. Results were interpreted based on predicted probabilities and factor combinations favoring normothermia. BWL and RT were correlated by thermal state using Spearman’s correlation.

Additionally, data from thermographic images (minimum, average, and maximum temperatures of the lacrimal area and ear) were correlated with RT data using Spearman’s correlation, including some rabbits from the thermal treatment T4.

### Metabolic response

The experimental unit for the biochemical data obtained from blood samples (i.e., HEM, GLU, CK, LDH, NEFAs, and CORT) was the cage, and the data were analyzed using a linear mixed model including thermal treatment (T1, T2, T3), available space (S121, S150, S191), and height (H20, H35), as well as their interactions, as fixed effects. The experimental session (1 to 9) was included as a random factor. Additionally, Pearson correlations were performed among these variables. A correlation matrix was produced using the “corrplot” package (16).

## Results

### Temperature and humidity conditions, initial body weight and actual space allowance

Temperature and relative humidity measurements recorded for each cage within the climate chamber are summarized in Table 1.

**Table 1:** Mean and standard deviation of ambient temperature and relative humidity in the climate chamber according to thermal treatment.

Thermal treatment	*n*	Temperature, °C	Relative humidity, %
T1	3	21.4 ± 1.7	68.6 ± 11.0
T2	3	25.9 ± 0.6	58.4 ± 4.3
T3	3	30.0 ± 0.5	55.8 ± 1.9
T4	1	33.5 ± 0.9	58.2 ± 3.5

*n*: number of replicates per thermal treatment.

Rabbits had a similar initial body weight (1.992 ± 0.041 kg) across thermal treatments (*p* = 0.346) and cages (*p* = 0.497). The actual space allowance for S191 was 191 ± 6 cm^2^/kg, for S150 was 150 ± 4 cm^2^/kg, and for S121 it was 121 ± 4 cm^2^/kg, corresponding to a stocking density of 53 ± 1.8, 67 ± 1.9, and 83 ± 2.8 kg/m^2^, respectively.

### Thermophysiological response

After 8 h of exposure to the different thermal treatments and movement restriction (combination of available space and cage height), variations in RT and BWL were observed in the rabbits (Table 2).

**Table 2:** Rectal temperature and body weight loss in fattening rabbits, calculated as the difference between body weight after 6 h of fasting and body weight after 8 h of exposure to different thermal treatments, in cages varying in space allowance and height.

Thermal treatment	Space allowance	Height	Rectal temperature, °C	Body weight loss, g
T1	S191	H20	38.9_e_	52.4_fg_
		H35	38.9_e_	50.0_g_
	S150	H20	39.2_cde_	55.6_fg_
		H35	39.1_cde_	66.2_cde_
	S121	H20	39.2_cde_	60.5_def_
		H35	39.1_cde_	61.2_def_
T2	S191	H20	39.1_de_	55.5_fg_
		H35	39.0_e_	57.5_efg_
	S150	H20	39.4_cde_	59.3_defg_
		H35	39.2_cde_	66.1_cde_
	S121	H20	39.6_bcd_	68.4_bcd_
		H35	39.3_cde_	67.3_cde_
T3	S191	H20	39.3_cde_	74.2_abc_
		H35	39.6_bc_	68.5_bcd_
	S150	H20	40.0_ab_	78.4_ab_
		H35	40.2_a_	73.1_abc_
	S121	H20	40.2_a_	77.3_ab_
		H35	40.4_a_	80.3_a_

Thermal treatments were: T1 (21.4 °C and 68.6% RH), T2 (25.9 °C and 58.4% RH), and T3 (30.0 °C and 55.8% RH). Space allowances were: S191: 191 cm^2^/kg; S150: 150 cm^2^/kg; and S121: 121 cm^2^/kg. The cage heights were: H20: 20 cm and H35: 35 cm. Different subscripts in the same column indicate that the means are statistically different between thermal treatments (*p* < 0.05).

While the average RT per rabbit was 39.8 °C (min: 35.6 °C; max: 41.5 °C), the average per cage was 39.4 °C (min: 38.0 °C; max: 41.0 °C). The statistical model used considered the cage as the experimental unit and was significant (R^2^m = 0.454, R^2^c = 0.778; *p* < 0.001). Thermal treatment tended to have an effect on RT (*p* = 0.080), available space had a significant effect on RT (*p* < 0.001), while cage height had no effect (*p* = 0.906). However, there was an interaction between thermal treatment and space allowance (*p* = 0.005), as well as between thermal treatment and cage height (*p* = 0.006). The model indicated that thermal treatment did not cause differences in RT between T1 and T2 (*p* = 0.588), nor between T1 and T3 (*p* = 0.237). However, space allowance did. Both S150 and S121 cages increased RT compared to S191 by the same magnitude (+0.4 °C; *p* = 0.01). Nevertheless, RT was similar between S150 and S121 (*p* = 0.897).

The interactions resulted in a tendency for increased RT in T2–S121 compared to T1–S121 (+0.3 °C; *p* = 0.054), and a significant increase in RT in T3–S121 compared to both T2–S121 (+0.4 °C; *p* = 0.022) and T1–S121 (+0.6 °C; *p* < 0.001). RT also increased in T3–H35 compared to T2–H35 (+0.4 °C; *p* < 0.01) and T1–H35 (+0.3 °C; *p* = 0.05).

When the average RT per cage was categorized by thermal state (hypothermia, normothermia, hyperthermia), the rabbits of 22 out of 162 cages were categorized as hypothermic, 116 as normothermic, and 24 as hyperthermic. Rabbits under T1 had a higher probability of hypothermia compared to T2 and T3 (*p* = 0.043). Among the space allowances tested, rabbits in S191 cages were more likely to experience hypothermia than those in S150 and S121, regardless of thermal treatment, while S150 and S121 showed similar results (Table 3). Cage height had no influence on the likelihood of hypothermia (*p* = 0.875), but it did increase the probability of hyperthermia under T3 conditions, especially in S150 and S121 (*p* < 0.037) (Table 3).

**Table 3:** The probability of rabbits experiencing hypothermia, normothermia, or hyperthermia according to thermal treatment, space allowance, and cage height.

Experimental treatments			Probability, %		
Thermal treatment	Space allowance	Height	Hipothermia	Normothermia	Hiperthermia
T1	S191	H20	40.6	59.4	0.0
		H35	41.1	58.9	0.0
	S150	H20	21.3	78.7	0.0
		H35	21.6	78.4	0.0
	S121	H20	26.4	73.6	0.0
		H35	26.8	73.2	0.0
T2	S191	H20	8.4	91.6	0.0
		H35	8.6	91.4	0.0
	S150	H20	3.5	96.5	0.0
		H35	3.6	96.4	0.0
	S121	H20	4.6	95.4	0.0
		H35	4.7	95.3	0.0
T3	S191	H20	11.9	78.9	9.1
		H35	11.6	75.3	13.1
	S150	H20	3.1	52.0	44.9
		H35	2.6	42.4	55.1
	S121	H20	2.3	29.5	68.2
		H35	1.8	21.9	76.3

Thermal treatments were: T1 (21.4 °C and 68.6% RH), T2 (25.9 °C and 58.4% RH), and T3 (30.0 °C and 55.8% RH). Space allowances were: S191: 191 cm^2^/kg; S150: 150 cm^2^/kg; and S121: 121 cm^2^/kg. The cage heights were: H20: 20 cm and H35: 35 cm. Hypothermia was defined as rectal temperature <38.6 °C, normothermia as 38.6–40.1 °C, and hyperthermia as >40.1 °C, in accordance with Kahn and Line (14).

Regarding BWL, the average per rabbit was 65.1 g (min: 3 g; max: 202 g), and the average per cage was 65.1 g (min: 31.6 g; max: 115.8 g). The statistical model considered the cage as the experimental unit and was significant (R^2^m = 0.319, R^2^c = 0.533; *p* < 0.001). Thermal treatment tended to have an effect on BWL (*p* = 0.074), space allowance had a significant effect on BWL (*p* < 0.001), while cage height had no effect (*p* = 0.578), and no significant interactions were found among the fixed factors (*p* > 0.05).

In terms of thermal treatment, BWL was similar between T1 and T2 (51 g vs. 57 g, respectively; *p* = 0.453). However, BWL increased in T3 compared to T1 (74 g vs. 51 g, +23 g; *p* < 0.01) and compared to T2 (74 g vs. 57 g, +17 g; *p* = 0.035). Rabbits in S121 cages had greater BWL compared to S191 (+8 g; *p* = 0.030) and tended to have greater BWL compared to S150 (+7 g; *p* = 0.074), regardless of the thermal treatment.

**Figure 1:**
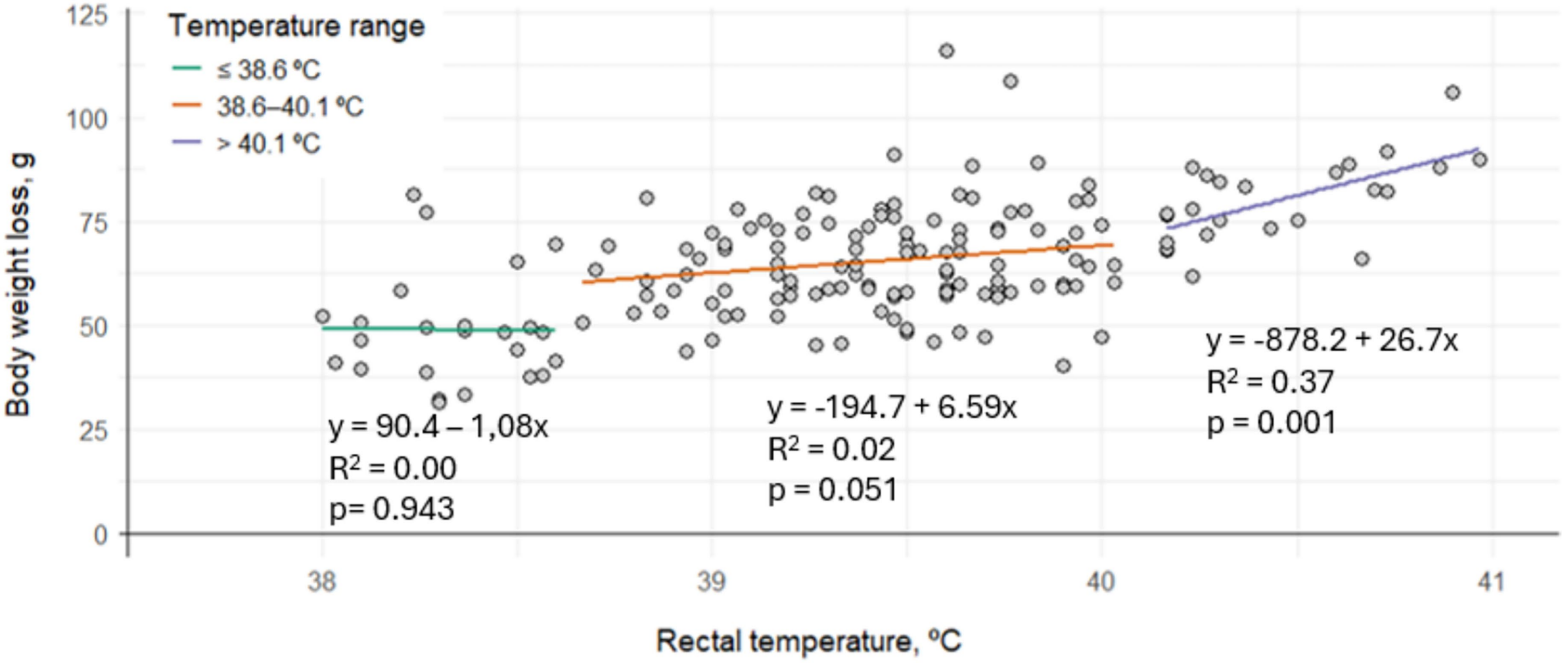
Linear correlation between the average rectal temperature and body weight loss per cage according to thermal state in fattening rabbits (hypothermia: ≤39.6 °C, normothermia: 38.5–40.1 °C, hyperthermia: >40.1 °C) subjected to 6 h of fasting and 8 h of exposure to different thermal treatments in cages varying in space allowance and height.

The relationship between thermal state and BWL is shown in Figure 1. No significant correlation between RT and BWL was observed in rabbits in hypothermia or normothermia (*p* > 0.05). However, in rabbits experiencing hyperthermia, BWL significantly increased with increasing RT (*p* = 0.001).

According to the statistical model, BWL varied significantly depending on the thermal state (*p* < 0.001). Cages with an average RT within the normothermic range had an average BWL of 65.2 g. In contrast, cages classified as hypothermic showed an average BWL of 54.4 g, which is 10.8 g less than the normothermic group. On the other hand, cages with average RT indicative of hyperthermia recorded an average BWL of 74.1 g, which is 8.9 g more than in normothermic rabbits.

**Figure 2:**
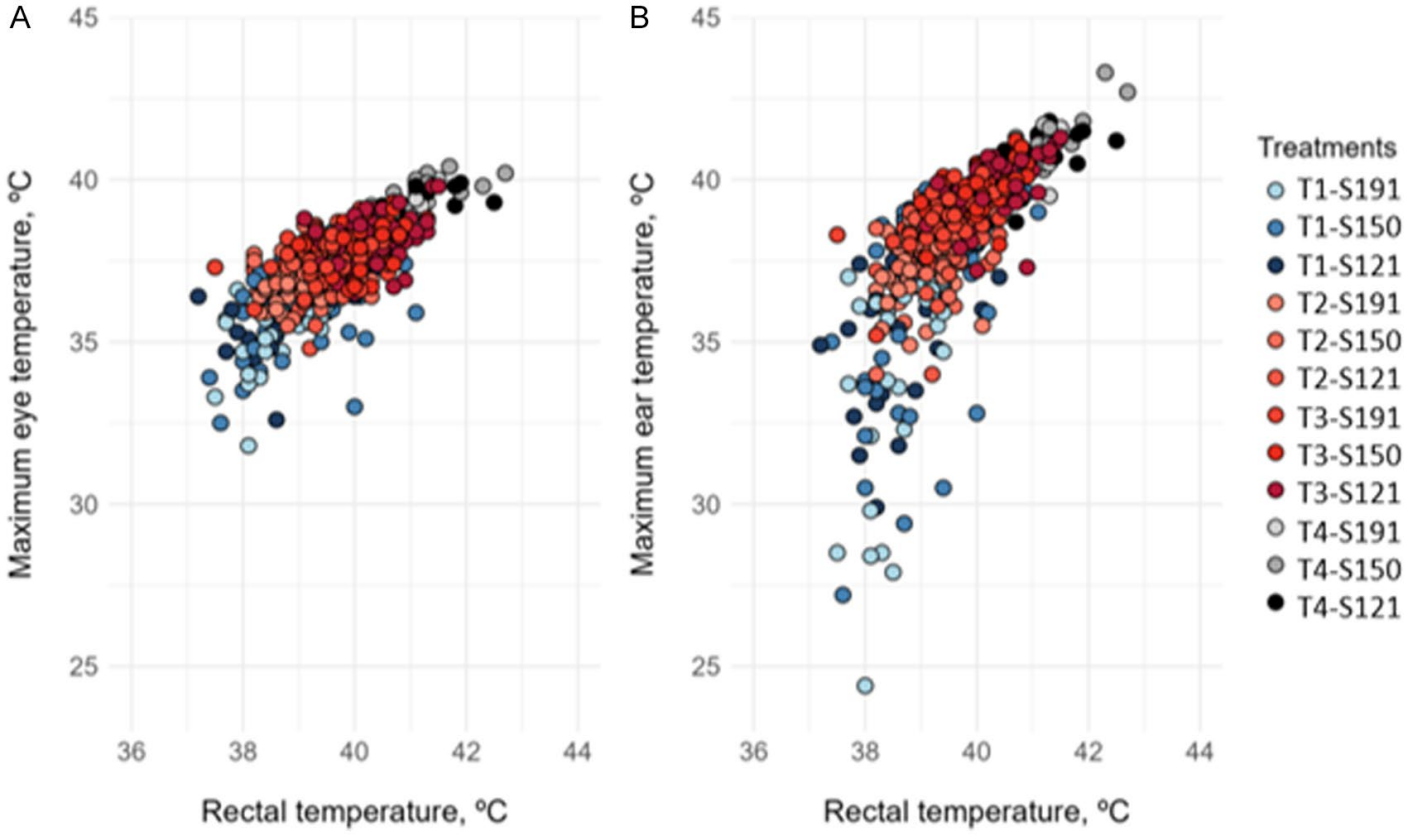
Correlation between **(A)** rectal temperature and maximum eye (lacrimal) temperature, and **(B)** rectal temperature and maximum ear temperature in fattening rabbits subjected to 6 h of fasting and 8 h of different thermal treatments in cages varying in available space and height. Treatments were: T1 (21.4 °C and 68.6% RH), T2 (25.9 °C and 58.4% RH), and T3 (30.0 °C and 55.8% RH), and T4: 33.5 °C and 58.2% RH. Available space: S191: 191 cm^2^/kg; S150: 150 cm^2^/kg; S121: 121 cm^2^/kg.

The minimum, maximum, and average temperatures of the lacrimal region and ear of the rabbits, obtained via thermographic camera, were correlated with RT. The strongest correlations were observed between the maximum temperature of the lacrimal region and ear and the rectal temperature. The plots representing these correlations are shown in Figure 2.

A positive and significant correlation was observed between RT and maximum lacrimal temperature (y = 0.514x + 20.42; r = 0.743; *p* < 0.001). Similarly, RT showed a positive and significant correlation with maximum ear temperature (y = 0.514x + 20.42; r = 0.704; *p* < 0.001). However, the correlation between RT and maximum ear temperature was weaker in rabbits from treatment T1(y = 0,155x + 33.43; r = 0,608; *p* < 0,001) compared to the combined treatments T2, T3, and T4 (y = 1,180x – 7.99; r = 0,742; *p* < 0,001).

### Metabolic response

The results of the biochemical analyses in blood samples are summarized in Table 4.

**Table 4:** Metabolic and stress markers in fattening rabbits subjected to 6 h of fasting and 8 h of different thermal treatments in cages varying in space allowance and height.

Thermal treatment	Space allowance	Height	HEM, %	GLU, mg/dL	LDH, U/L	CK, U/L	NEFAs, mmol/L	CORT, ng/mL
T1	S191	H20	35.7	133_cd_	228	699	0.510_abc_	19.1
		H35	35.6	130_d_	335	957	0.597_a_	20.7
	S150	H20	36.2	138_bcd_	349	1,043	0.432_abc_	18.8
		H35	36.1	133_cd_	256	918	0.547_ab_	21.2
	S121	H20	35.1	144_abcd_	298	1,259	0.430_abc_	19.3
		H35	35.3	138_bcd_	207	773	0.532_ab_	20.8
T2	S191	H20	35.6	138_bcd_	917	1,098	0.433_abc_	18.4
		H35	35.3	132_cd_	463	1,121	0.438_abc_	18.8
	S150	H20	35.7	133_cd_	380	937	0.523_abc_	20.2
		H35	35.7	149_abcd_	643	1,042	0.380_bc_	15.2
	S121	H20	35.3	155_abc_	302	1,086	0.399_bc_	16.8
		H35	35.5	133_cd_	474	1,155	0.542_ab_	19.5
T3	S191	H20	36.0	143_abcd_	240	933	0.338_c_	21.5
		H35	35.2	141_abcd_	334	1,152	0.422_abc_	18.6
	S150	H20	34.8	155_abc_	231	937	0.420_abc_	20.0
		H35	35.8	147_abcd_	352	1,049	0.382_bc_	19.0
	S121	H20	35.4	163_a_	248	1,260	0.444_abc_	16.9
		H35	35.5	156_ab_	368	1,008	0.367_bc_	18.6

HEM: hematocrit; GLU: glucose; LDH: lactate dehydrogenase; CK: creatine kinase; NEFAs: non-esterified fatty acids; CORT: corticosterone. T1: 20 °C and 50% RH; T2: 25 °C and 50% RH; T3: 30 °C and 50% RH. S191: 191 cm^2^/kg; S150: 150 cm^2^/kg; S121: 121 cm^2^/kg; H20: 20 cm; H35: 35 cm. Different subscripts in the same column indicate that the means are statistically different between thermal treatments (*p* < 0.05).

HEM and LDH remained stable across thermal treatments, available space, and cage height. The average HEM was 35.5% (min: 32.4%; max: 38.2%), while the average LDH was 405 U/L (min: 171 U/L; max: 2497 U/L). Regarding HEM, the mixed model used was significant (R^2^m = 0.003, R^2^c = 0.349; *p* < 0.001). However, HEM was not influenced by thermal treatment (*p* = 0.972), available space (*p* = 0.625), nor cage height (*p* = 0.770). No interaction was found among these variables (*p* > 0.05). Similarly, for LDH, the model was also significant (R^2^m = 0.101, R^2^c = 0.289; *p* < 0.001), but it was not affected by thermal treatment (*p* = 0.679), available space (*p* = 0.359), or cage height (*p* = 0.292). No interactions between variables were found (*p* > 0.05).

The average GLU was 142 mg/dL (min: 117 mg/dL; max: 181 mg/dL). The statistical model for GLU (R^2^m = 0.203, R^2^c = 0.348; *p* < 0.001) showed that GLU was not affected by thermal treatment (*p* = 0.200) but was influenced by space allowance (*p* = 0.002) and tended to be affected by cage height (*p* = 0.070), resulting in higher GLU values in S111 and a tendency for higher GLU in H20.

The average CK was 1,024 U/L (min: 567 U/L; max: 1935 U/L). CK (R^2^m = 0.092, R^2^c = 0.329; *p* < 0.001) was only affected by the interaction between available space and cage height. CK levels were similar across thermal treatments and cage types except for an increase in S121-H20 (+271 U/L; *p* = 0.030). However, posthoc tests showed no significant differences between tested combinations (*p* > 0.05), as shown in Table 4.

The average NEFAs was 0.452 mmol/L (min: 0.243 mmol/L; max: 0.807 mmol/L). The model (R^2^m = 0.191, R^2^c = 0.392; *p* < 0.001) showed that NEFAs were only affected by thermal treatment. Specifically, both T3 and T2 tended to decrease NEFAs by 28% compared to T1 (*p* < 0.010).

The average CORT was 19.3 ng/mL (min: 12.1 ng/mL; max: 33.0 ng/mL). The model (R^2^m = 0.129, R^2^c = 0.456; *p* < 0.001) revealed an interaction between thermal treatment, available space, and cage height (*p* = 0.020). However, posthoc comparisons showed no significant differences between combinations of these variables, as observed in Table 4.

**Figure 3:**
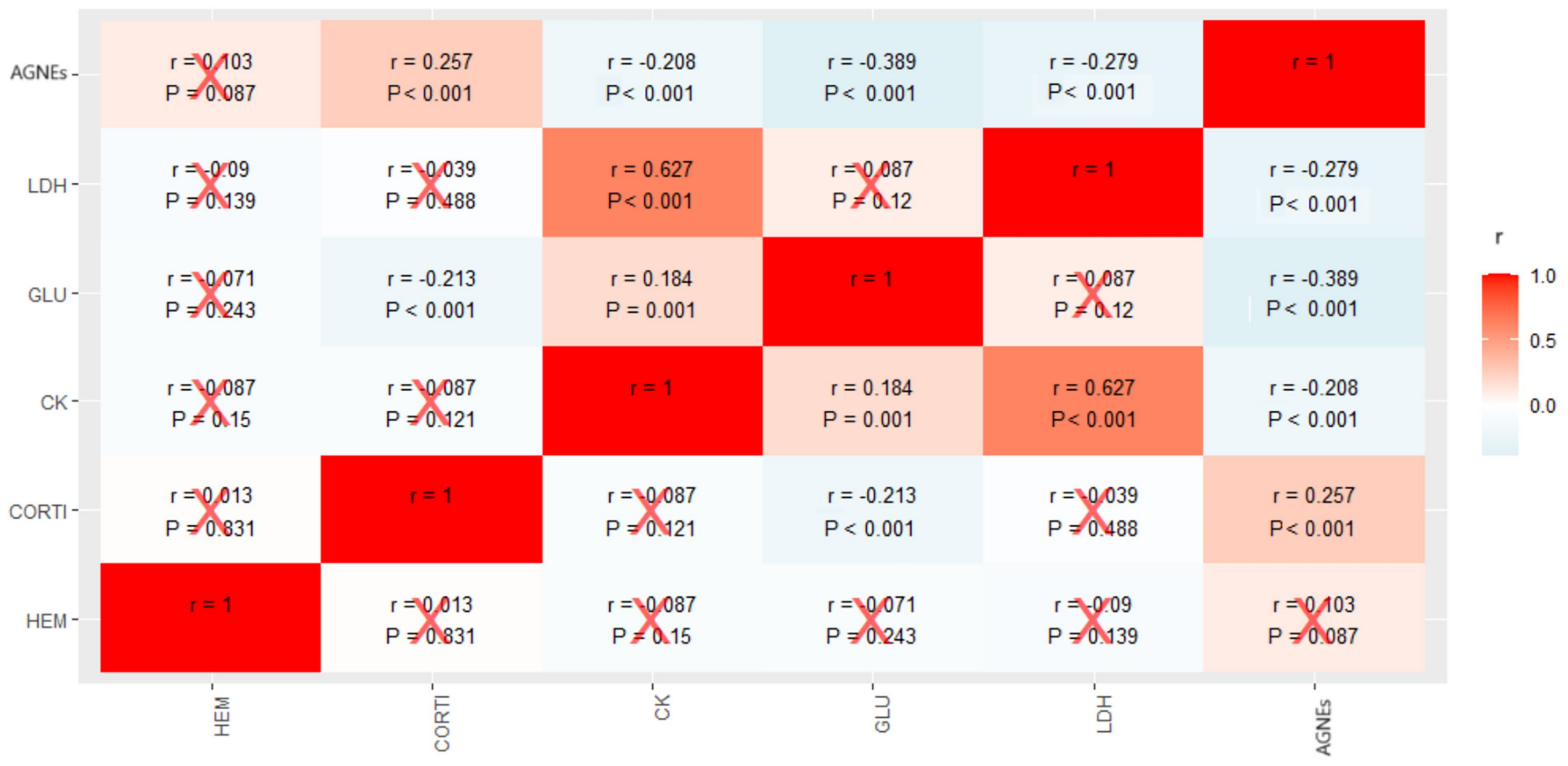
Heatmap of the correlations between metabolic markers in blood plasma of fattening rabbits subjected to 6 h of fasting and 8 h of exposure to different thermal treatments in cages varying in available space and height. HEM: hematocrit; CORT: corticosterone; CK: creatine kinase; GLU: glucose; LDH: lactate dehydrogenase; NEFAs: non-esterified fatty acids. The values in the table are Spearman correlation coefficients (r) and *p*-values. Red crosses indicate non-significant correlations (*p* > 0.05).

The relationships between markers are shown in Figure 3. A positive and statistically significant correlation was found between CORT and NEFAs, between CK and LDH, and between CK and GLU. Negative and statistically significant correlations were also observed between GLU and CORT, GLU and NEFAs, CK and NEFAs, and LDH and NEFAs.

## Discussion

### Temperature and humidity conditions, initial live weight, and available space

Regarding the temperature and humidity conditions recorded by the sensors in the climatic chamber, the relative humidity was higher than expected during the experimental sessions of T1 due to the chamber’s inability to remove ambient relative humidity. This was because two of the three experimental sessions coincided with rainy days. Nevertheless, the temperature and humidity conditions achieved in the climatic chamber for each thermal treatment were similar to those initially intended in the study except for T1 that was higher. The rabbits subjected to this study started with a similar initial body weight equivalent to commercial weight in Spain.

At a space allowance of 191 cm^2^/kg, the rabbits had enough space for all of them to lie down simultaneously (Contreras-Jodar, unpublished). However, at 150 and 121 cm^2^/kg, not all rabbits could lie down at the same time and, if they did, some degree of piling occurred. In H35 cages, the rabbits had enough height to sit on their hind legs and hold their ears upright if desired, allowing for a more natural and less restrictive posture (height being the more demanding factor). In contrast, H20 cages did not offer sufficient height for the animals to remain seated but did allow them to lie down comfortably (Contreras-Jodar, unpublished).

### Thermophysiological response

The thermal comfort zone of rabbits is between 13 and 20 °C (17). Unlike other mammals, their thermoregulation capacity depends largely on behavioral strategies rather than physiological mechanisms. In their natural environment, rabbits seek refuge in underground burrows during the hottest hours, taking advantage of the thermal stability of these communal systems, which are predominantly dug by females, consistent with the species’ social organization. Inside these galleries, microenvironmental conditions remain within a thermoneutral range, facilitating physiological processes such as cecotrophy while they wait for temperatures to drop to go out and feed at dusk (11).

Under intensive production and transport conditions, rabbits do not have the possibility to regulate their temperature through these behaviors, exposing them to adverse climatic variations on the farm, especially during capture and transport. When environmental temperature rises, these animals activate different mechanisms to dissipate heat and restore thermal balance. Among the most common physiological responses in mammals are panting and sweating, mechanisms that favor heat loss by evaporation. However, in rabbits, these processes are inefficient because they have a limited number of functional sweat glands and a reduced capacity for heat dissipation through panting (18).

To compensate for this limitation, rabbits resort to alternative strategies, such as increasing blood flow to the ear, facilitating heat dissipation by convection (11), or postural modifications like lying down on the floor of the transport container to maximize the exposed body surface and enhance heat loss (19). However, the effectiveness of these mechanisms is compromised in overcrowded conditions, where space restriction reduces the animal’s thermoregulation capacity (19).

When an animal’s thermoregulatory capacity is exceeded, its body temperature increases. Therefore, monitoring body temperature is key to detecting heat stress in animals, as it acts as a sensitive indicator of the organism’s adaptive capacity (20). Thus, increased body temperature reflects the animal’s inability to cope with heat stress and its vulnerability to its negative effects (21). In rabbits, normal rectal temperature ranges between 38.6 and 40.1 °C (14), so values below 38.6 °C indicate hypothermia, while temperatures above 40.1 °C suggest hyperthermia. Both conditions can seriously compromise animal welfare and, in extreme cases, be lethal.

In the present study, RT was measured in rabbits after exposure to different thermal treatments and movement restrictions (different combinations of space allowance and cage height). Some cages had rabbits with an average RT below 38.6 °C, suggesting risk of hypothermia, and others with an average above 40.1 °C, related to risk of hyperthermia.

Cages where rabbits were in risk of hypothermia mainly corresponded to T1 and, to a lesser extent, T2 and T3. One possible explanation for this is that urination by some rabbits wet the fur of their cage mates and the accumulated moisture may have favored excessive body heat loss, thus contributing to the decrease in RT. These findings agree with those reported by Finzi (11), who observed that wetting the bodies of rabbits, except the head, caused a 1 °C reduction in RT, highlighting the impact of fur moisture on the species’ thermoregulation loss.

Cages where rabbits experienced risk of hyperthermia corresponded only to the T3 sessions, with the condition intensifying as space allowance decreased and cage height increased. The reduction in space allowance, which equates to higher stocking density and thus closer contact between individuals, contributed to the increase in RT due to the accumulation of metabolic heat. Under these conditions, body heat dissipation is compromised, since heat exchange with the environment is limited and rabbits are exposed to radiant heat from their cage mates. As a consequence, the T4 thermal treatment (i.e., 33.5 °C and 58% RH) had to be stopped after 5 h, because heat stress severely compromised the survival of rabbits in S121 and S150, especially in the H35 cages.

On the other hand, the increase in cage height also negatively impacted the rabbits’ RT. In the H35 cages, rabbits tended to pile on top of each other, a strategy that may be related to their natural motivation to seek a thermally more favorable environment, to show hierarchy or just to look for the company on conspecifics in a new environment. In this arrangement, rabbits on the top layer remained in better thermal conditions, likely due to higher airflow and less physical contact. In contrast, individuals in the lower layer experienced greater heat stress, unable to dissipate heat from the environment and from the bodies of their conspecifics.

Finzi (11) observed that an increase in ambient temperature from 25 °C to 30 °C caused a 0.4 °C increase in rabbits’ body temperature. Raising the temperature from 30 °C to 35 °C resulted in a 1.9 °C increase. This suggests that above 30 °C, rabbits’ thermoregulatory mechanisms lose efficiency rapidly, making them particularly vulnerable to heat stress conditions. However, that study did not consider the possible additive effect of body heat generated by other rabbits, nor its interaction with cage height. The limited thermal tolerance of rabbits can result in significant mortality when ambient temperature reaches 35 °C and is maintained for hours, as observed both in the present study and in Finzi’s findings (11).

In this study, cages remained static inside a climatic chamber, where no airflow was applied except the system’s minimal ventilation. This minimal ventilation may have increased the risk of heat stress by not favoring efficient body heat dissipation, while it could have reduced the risk of cold stress, since the lack of air circulation may have increased heat loss in low-temperature conditions. In comparison, during rabbit transport by truck, airflow generated by natural ventilation could help relieve heat stress but might also increase the risk of cold stress.

Unlike rabbits in T1 and T2, all rabbits in T3 were found with dry fur at the end of the experimental session, although some showed clear signs of having been urinated on at some point. The high temperature in T3 may have favored a more rapid evaporation of urine. In addition, rabbits exposed to T3 may have reduced their urine production as a physiological strategy to conserve water and minimize dehydration, as animals prioritize fluid retention to maintain water balance under these conditions.

It is worth noting that, although rabbits’ fur were qualitatively assessed, there was not scored according to the level of wetness. However, the presence of wet animals seemed urination as a common behavior during rabbit transport (19, 22). This phenomenon often occurs when animals from different cages that have not previously shared space are grouped together. Under such circumstances, the change of environment and interaction with unfamiliar individuals can induce behavioral responses such as territorial marking through urine (22). This behavior is more frequently observed during the loading process of animals onto transport vehicles, particularly when cages are stacked in towers (19). Urinary marking serves an important social function, as it is a mechanism used by rabbits to establish dominance hierarchies within the group (22). It should also be noted that under real transport conditions, rabbits may dry more quickly due to the air flow generated by truck movement. However, this airflow may also reduce the animals’ effective temperature, potentially aggravating cold stress, especially in wet animals. Therefore, the impact of urination on the rabbit’s thermoregulatory capacity remains uncertain. During live animal transport, rabbits remain without access to water or food, which inevitably results in BWL.

Initially, this BWL is due to emptying of intestinal contents, loss of body water (through respiration and urination), and mobilization of energy reserves. However, heat stress causes an increased mobilization of these reserves, contributing to greater BWL, as observed in numerous studies and across different animal species (23–26). This phenomenon is caused by alterations in carbohydrate, lipid, and protein metabolism after absorption, as well as coordinated changes in energy supply and utilization across various tissues (27). Therefore, BWL can also be a sensitive indicator of heat stress in transported rabbits.

On the other hand, rabbits experiencing hypothermia lost significantly less body weight than normothermic and hyperthermic individuals. This is likely because evaporative water loss processes are presumed to be reduced in hypothermic states, contributing to lower overall BWL. Thus, although hypothermia represents a physiological risk state, its lower impact on energy reserve mobilization and dehydration could explain the reduced BWL observed in these animals.

In the present study, all rabbits experienced BWL after remaining 8 h in the thermal chamber, initially as a result of energy reserve mobilization induced by 6 h of fasting prior to the transport simulation plus an additional 8 h of fasting in the thermal chamber. However, the magnitude of BWL was not uniform among all rabbits (ranging from 51 to 80 g; equivalent to a BWL of 2.6 to 4.0% of live weight). BWL was similar across all T1 and T2 cages regardless of space allowance and cage height. BWL increased in T3 regardless of space allowance and cage height, with numerically greater BWL in cages with less available space. Unlike RT, cage height (H35) had no effect on BWL compared to H20 cages. Previous studies noted that rabbits lost 56.5 g after 7 h of transport (10), while others reported a BWL of 3.5% of live weight after 6 h of transport, suggesting that the thermal chamber simulation had a similar effect on BWL as commercial transports.

The high correlation between RT and temperature recorded at the lacrimal and ear regions using infrared thermography supports the use of this technique as a non-invasive method to assess thermal stress in rabbits. In our study, the lacrimal region correlated better with RT than the auricular region. These findings align with those reported by Finzi (11), who observed that thermal variability of the ear decreases with increasing ambient temperature, suggesting progressive use of passive heat dissipation mechanisms when thermoregulation is compromised. This process is associated with evident peripheral vasodilation and postural adjustment of the ears, which adopt a lateral position to reduce muscular effort and minimize thermal radiation interference with other body parts (11). The ability of rabbits to modulate these mechanisms underscores the relevance of infrared thermography in monitoring heat stress and animal welfare.

From an applied perspective, thermography offers great potential for monitoring heat stress during live animal transport, enabling rapid identification of individuals at risk and optimization of management strategies to minimize heat impact. However, its accuracy may be affected by factors such as ambient humidity and airflow speed. Despite these limitations, combining thermography with other physiological indicators can significantly improve the evaluation of animal welfare under heat stress conditions.

### Metabolic response

In the present study, various metabolic and stress markers were analyzed, including hematocrit (HEM), glucose (GLU), lactate dehydrogenase (LDH), creatine kinase (CK), advanced glycation end products (AGEs), and corticosterone (CORT), with the objective of assessing the impact of heat stress and movement restriction on the welfare of fattening rabbits.

Mixed-effects models revealed a notable difference between the marginal coefficient of determination (R^2^m) and the conditional coefficient of determination (R^2^c) for several physiological markers. The R^2^m represents the proportion of variance explained solely by the fixed effects (i.e., thermal treatment, available space, and cage height), whereas the R^2^c includes both fixed and random effects (in this case, the experimental day, ranging from day 1 to day 9). Each experimental day involved 90 rabbits, representing different batches originating from the farm.

The substantial differences observed between R^2^m and R^2^c, such as for HEM (R^2^m = 0.003; R^2^c = 0.349) or LDH (R^2^m = 0.101; Rc = 0.289), suggest that most variability in these markers is not explained by the manipulated experimental factors but rather by random effects included in the model. This pattern was also observed for other biomarkers such as CK (R^2^m = 0.092; R^2^c = 0.329), AGNEs (R^2^m = 0.191; R^2^c = 0.392), and CORT (R^2^m = 0.129; R^2^c = 0.456), reinforcing the hypothesis that individual or group variability (e.g., among animals or batches) has a stronger influence than systematic treatment effects. These results highlight the importance of including random effects in the models, particularly when involving multiple sampling days or animals from different cohorts.

HEM is an indicator of the percentage of blood volume occupied by red blood cells and plays an important role in the assessment of dehydration. Under dehydrated conditions, plasma volume decreases, leading to a concentration of the cellular components of the blood and, consequently, an increase in HEM (28). However, in the present study, HEM was similar across all thermal treatments, space allowances and heights (range 34.8 to 36.2%), suggesting that rabbits were able to maintain a consistent hydration status despite the different experimental conditions. This may be explained by the rabbit’s ability to reduce urinary excretion and recycle water contained in muscle tissue during protein degradation, thereby optimizing the use of available water resources. Similar results have been reported in Giant breed rabbits during transport under different thermal and space availability conditions, where no significant variations in HEM were observed (8). However, in non-transported rabbits, the average HEM was 32.4%, suggesting a higher hydration state compared to those subjected to transport (8). Likewise, Liste et al. (9) reported that HEM did not vary in hybrid rabbits regardless of transport duration (1 or 7 h), season (summer: 27 °C, 48% RH; winter: 12 °C, 63% RH), or container position within a truck with a space allowance of 367 cm^2^ (equivalent to a density of 60 kg/m^2^).

On the other hand, GLU is one of the main energy sources of the organism, and its blood concentration is regulated by a balance between hormones such as insulin and glucagon and it is affected by stress hormones, like CORT. In this study, GLU ranged between 130 and 166 mg/dL and was affected by a three-way interaction among thermal treatment, space allowance, and cage height. It was observed that the combination T3, S121, and H20 led to an increase in blood GLU, although *post hoc* comparisons showed that GLU levels were similar among T2-S150, T2-S121, T3-S191, T3-S150, and T3-S121, and higher than in the other combinations, regardless of cage height. This effect is due to activation of the sympathetic nervous system under heat stress conditions, which promotes the release of catecholamines and CORT, increasing gluconeogenesis and reducing peripheral tissue uptake of GLU. These results are consistent with previous studies showing increased GLU levels in rabbits transported in confined spaces and under summer conditions (8).

Rabbits transported in summer (thermal range 29–45 °C) showed higher GLU levels than those transported in winter (thermal range 7–23 °C) and those traveling in lower space allowance had higher GLU concentrations than those with more space (8). Similarly, in 1.2 kg live-weight rabbits, plasma GLU concentrations increased with ambient temperature (29). Consequently, it can be inferred that the combination of pre-transport fasting, heat stress caused by thermal treatments during transport, and additional heat stress generated by conduction with the body temperature of other rabbits in the same cage (which increases as space per animal decreases) raises plasma GLU levels.

The lack of changes in CORT levels despite increased GLU and heat stress could indicate several possibilities. Rabbits might have developed a heat adaptation response after 8 h in the climatic chamber, which could reduce hypothalamic–pituitary–adrenal (HPA) axis activation and limit CORT release as occur in other mammals (30). Alternatively, the GLU increase could result from alternative hormonal mechanisms, such as catecholamine release (adrenaline and noradrenaline), which elevate blood GLU without raising CORT.

LDH is an enzyme that plays an important role in GLU metabolism, especially under anaerobic conditions or when energy demand exceeds the capacity of the aerobic system. Under heat stress conditions, cellular metabolism undergoes adaptive changes to cope with increased temperature and the resulting disruption of homeostasis (31). The finding that LDH levels were similar among thermal treatments and cages with different levels of movement restriction, could be explained by the fact that no significant cell damage was produced.

CK, on the other hand, is a key enzyme in muscle energy metabolism and a sensitive marker of muscle damage. In the present study, no differences in CK were found between thermal treatments, but differences were observed between cages with varying space availability. The smaller the available space, the higher the plasma CK levels. This could be due to greater muscle damage associated with movement restriction and/or heat radiation from other rabbits within the cage, rather than a direct effect of the thermal treatment. The significant positive correlation between CK and LDH suggests that both enzymes are responding in a synchronized manner.

NEFAs are lipids circulating in the blood that originate from the lipolysis of triglycerides stored in adipose tissue. They serve as a key energy source for many tissues, especially during fasting or metabolic stress (32). In the present study, the lowest NEFAs levels were recorded in rabbits exposed to T2 and T3. This could suggest reduced NEFAs mobilization under these conditions or, alternatively, increased utilization of NEFAs as an energy source, accompanied by enhanced catabolism towards glucose. The negative correlation found between NEFAs and LDH/GLU may suggest that, under hypothermia/normothermia conditions, rabbits may mobilize adipose tissue and utilize NEFAs as an energy source while in hyperthermia, glucose and gluconeogenic precursors become the primary energy sources (27, 33, 34).

### Limitations

The actual space allowances tested during the experiment differed from the initially planned target values (111, 143, and 182 cm^2^/kg). Because the rabbits’ final body weights were lower than expected, the achieved space allowances were correspondingly higher (121, 150, and 191 cm^2^/kg). As a result, we were unable to test the specific target space allowances originally intended.

This study, conducted in a climatic chamber, allowed us to simulate different temperature conditions, space allowances, and cage heights to evaluate their impact on commercial-weight rabbits. However, it presents certain limitations when compared to commercial transport by truck, where animals are exposed to multiple additional factors. In transport trucks, rabbits are typically allocated in stacked containers, which may affect air circulation differently than in the climatic chamber, where animals were allocated in single-tier cages.

In real-world scenarios, elements such as noise, vibrations, acceleration and braking, as well as air turbulence generated by vehicle speed, may interact with temperature and space, affecting rabbits’ responses in ways that differ from those observed in a controlled environment. Furthermore, in a moving vehicle, airflow varies depending on speed, type of transport container, and truck design, which influences thermal sensation and heat distribution, factors that cannot be replicated in a climatic chamber.

Additionally, the loading and unloading process itself can generate stress, which was not evaluated in this study. By isolating specific variables, it is also possible that some complex interactions occurring during commercial transport were not captured, where the combination of multiple factors may trigger different physiological responses. Despite these limitations, the use of a climatic chamber enables precise experimental control and facilitates the interpretation of the individual effects of each variable. However, these aspects should be considered when extrapolating the results to commercial transport conditions.

## Conclusion

Space allowance and cage height influences the thermophysiological and metabolic responses of fattening rabbits depending on the thermal conditions.

At ambient temperatures exceeding 30 °C and relative humidity of 56–58% and under minimal air velocity, a minimum space allowance of 191 cm^2^/kg (maximum stocking density of 53 kg/m^2^) combined with a transport container height of 20 cm (as opposed to the 35 cm tested) mitigate the risk of hyperthermia. This limitation in height appears to reduce vertical piling of animals and, consequently, the differential exposure to negative thermal gradients, particularly for rabbits placed in lower cage levels, which are more prone to heat accumulation. In addition, at lower ambient temperatures, no benefit is observed from having 35 cm instead of 20 cm container height. On the other hand, at ambient temperature from 20 to 25 °C, space allowance between 121 and 150 cm^2^/kg (67 and 83 kg/cm^2^) decreased the risk of animals suffering hypothermia compared to 191 cm^2^/kg (53 kg/m^2^), which may be caused by moisture accumulation in the fur due to urine.

These findings should be validated under commercial transport conditions, where additional factors such as ventilation and airflow dynamics may modify the thermal sensation. Integrating all relevant variables will be essential to establish practical recommendations for future regulations on rabbit welfare during transport.

On the other hand, thermographic imaging proved to be a reliable, non-invasive method for assessing thermal stress in rabbits. The lacrimal region shows a strong correlation with rectal temperature across all thermal states (hypothermia, normothermia and hyperthermia), making it a consistent indicator. In contrast, the auricular region only correlates well under heat stress, likely due to increased blood flow for heat dissipation. These findings support the use of lacrimal thermography for routine welfare monitoring during transport.

## Data Availability

The raw data supporting the conclusions of this article will be made available by the authors, without undue reservation.
